# The impact of a regionally based translational cancer research collaborative in Australia using the FAIT methodology

**DOI:** 10.1186/s12913-024-10680-2

**Published:** 2024-03-11

**Authors:** Christine L. Paul, Nicole M. Verrills, Stephen Ackland, Rodney Scott, Susan Goode, Ann Thomas, Sarah Lukeman, Sarah Nielsen, Judith Weidenhofer, James Lynam, Elizabeth A. Fradgley, Jarad Martin, Peter Greer, Stephen Smith, Cassandra Griffin, Kelly A. Avery-Kiejda, Nick Zdenkowski, Andrew Searles, Shanthi Ramanathan

**Affiliations:** 1 School of Medicine and Public Health, University of Newcastle, Newcastle, NSW Australia; 2 Hunter Medical Research Institute, Newcastle, NSW Australia; 3 School of Biomedical Sciences and Pharmacy, University of Newcastle, Newcastle, NSW Australia; 4 Calvary Mater Hospital Newcastle, Newcastle, NSW Australia; 5 Hunter New England Local Health District, Newcastle, NSW Australia

**Keywords:** Cancer research, Translational research, Impact assessment, Rural and regional health, Consumer involvement

## Abstract

**Background:**

Translating research, achieving impact, and assessing impact are important aspirations for all research collaboratives but can prove challenging. The Hunter Cancer Research Alliance (HCRA) was funded from 2014 to 2021 to enhance capacity and productivity in cancer research in a regional centre in Australia. This study aimed to assess the impact and benefit of the HCRA to help inform future research investments of this type.

**Method:**

The Framework to Assess the Impact from Translational health research (FAIT) was selected as the preferred methodology. FAIT incorporates three validated methodologies for assessing impact: 1) Modified Payback; 2) Economic Analysis; and 3) Narrative overview and case studies. All three FAIT methods are underpinned by a Program Logic Model. Data were collected from HCRA and the University of Newcastle administrative records, directly from HCRA members, and website searches.

**Results:**

In addition to advancing knowledge and providing capacity building support to members via grants, fellowships, scholarships, training, events and targeted translation support, key impacts of HCRA-member research teams included: (i) the establishment of a regional biobank that has distributed over 13,600 samples and became largely self-sustaining; (ii) conservatively leveraging $43.8 M (s.a.$20.5 M - $160.5 M) in funding and support from the initial $9.7 M investment; (iii) contributing to clinical practice guidelines and securing a patent for identification of stem cells for endometrial cell regeneration; (iv) shifting the treatment paradigm for all tumour types that rely on nerve cell innervation, (v) development and implementation of the world’s first real-time patient treatment verification system (Watchdog); (vi) inventing the effective ‘EAT’ psychological intervention to improve nutrition and outcomes in people experiencing radiotherapy for head and neck cancer; (vi) developing effective interventions to reduce smoking rates among priority groups, currently being rolled out to disadvantaged populations in NSW; and (vii) establishing a Consumer Advisory Panel and Consumer Engagement Committee to increase consumer involvement in research.

**Conclusion:**

Using FAIT methodology, we have demonstrated the significant impact and downstream benefits that can be achieved by the provision of infrastructure-type funding to regional and rural research collaboratives to help address inequities in research activity and health outcomes and demonstrates a positive return on investment.

**Supplementary Information:**

The online version contains supplementary material available at 10.1186/s12913-024-10680-2.

## Background

Cancer is a major cause of disability and death worldwide [1]. The Australian Burden of Disease Study identified cancer as the leading cause of disease burden in Australia in 2018, accounting for 18% of the total burden [2]. Although cancer outcomes in New South Wales (NSW), the most populous state in Australia, are among the best in the world, outcomes remain poorer for people living in regional and remote areas compared to people living in the capital, Sydney. For example, the Hunter New England (HNE) region of NSW has 1.05 times the age-standardised cancer rate and 1.11 times the standardised mortality ratio compared to the state average [3]. Reasons for this include poorer access to high-quality cancer care, higher rates of cancer risk factors such as smoking and alcohol consumption, and higher rates of low survival cancers in these regions [4, 5].

Research targeting cancer risk, cancer treatment and cancer care in non-metropolitan populations is needed to address this inequity in cancer outcomes. The likelihood of the successful translation of effective research outcomes that address the needs and outcomes of specific populations into clinical practice is higher if that research is co-designed with relevant stakeholders and accounts for regional capacity and needs [6]. In addition, building a strong research culture around and within regional and rural health services can facilitate improved cancer care and cancer outcomes [7]. However, undertaking translation-focussed, co-designed research in rural and regional areas is challenging given high workloads, the need for a ‘critical mass’ in research [8] and poorer access to research infrastructure such as biobanks, research training, capacity building and research leadership opportunities, compared to metropolitan centres.

Providing research skills and supportive infrastructure is not cost-free. The rising cost of providing healthcare and rising demand driven by an ageing population, exacerbated by the recent pandemic, have heightened the need for Australian health services and research funders to encourage greater translation of effective research evidence into policy and practice as a means to optimise the impact from that research, and hence, the returns on research investments [9–13].

Considering these issues, the Cancer Institute of NSW (CINSW) invested in translational cancer research centres (TCRCs) to increase cancer research capacity, productivity, and translation in NSW; with the mission of improving cancer outcomes and creating a competitive global hub of excellence in translational cancer research in NSW. CINSW is the State’s cancer control agency, providing the strategic direction for cancer control in NSW to promote better cancer prevention, early detection, diagnosis, treatment, and care. The CINSW invested $AUD8.75 million between 2014 and 2021 to support a TCRC in the HNE region of NSW. The HNE region (including Newcastle where many researchers were based) includes metropolitan, inner-regional, outer regional areas along with a small portion classified as remote. As such, the population is somewhat comparable to much of the non-capital-city areas along eastern Australia. The Hunter Cancer Research Alliance (HCRA) was the sole TCRC based outside metropolitan Sydney. HCRA was an umbrella organisation which drew together four existing cancer research programs operating throughout the HNE region: the Hunter Medical Research Institute’s (HMRI) Cancer Research Program; University of Newcastle’s (UoN) Priority Research Centre in Cancer Research, Innovation and Translation; Hunter Translational Cancer Research Unit; and the Clinical Cancer Research Network. As a multidisciplinary and multi-institutional alliance, the HCRA brought together over 270 researchers and clinicians from HMRI, UoN and the Hunter New England Local Health District (HNELHD).

The impact from research funding is typically documented in academic terms such as papers produced, conferences attended, or additional grants leveraged from the original seed funding. In addition to this, CINSW sought evidence of wider, non-academic, impacts from the research it supported, where impacts would include research capacity and capability building, consumer involvement in research, research evidence that translated into policy and practice, and community benefit. The CINSW support of the HCRA ended in 2021. This study aimed to capture and report the cost and the impact from the CINSW investment in research channelled through the HCRA.

Although the collaborative and synergistic nature of this type of research investment poses greater challenges for attributing specific impacts to the investment (in this case HCRA), it is still a worthwhile endeavour to inform future major investments of this kind. There are a range of frameworks that can be used to estimate research impact [14–18]. The framework selected for this study was the Framework to Assess the Impact from Translational health research (FAIT), which was developed by a team of health economists and researchers based at HMRI [15]. FAIT was selected due to its focus on health and medical research; its ability to be used retrospectively and prospectively, and its comprehensive methodological foundation of three approaches to impact assessment- that is metrics, economic assessment, and a narrative. The FAIT approach was able to be supported by data collected by HCRA staff. The Health Economics and impact team at HMRI were engaged to provide independent, expert support and guide the application of the FAIT to HCRA.

This study aimed to assess the impact and benefit of the HCRA to help inform future decisions about health research investment.

## Methods

### Setting and participants

The setting for the impact assessment was the operations office of the HCRA, based at HMRI in Newcastle, Australia. HCRA membership included cancer researchers, clinicians and consumers based in the HNE and Central Coast region of NSW. The region includes two regional Universities (UoN, where the majority of HCRA researchers are affiliated, and the University of New England), a single research institute (HMRI) and a single Local Health District (HNELHD) which serves over 850,000 people living in regional and rural areas covering over 130,000 km^2^ (see Supplementary Figure). HCRA activities were coordinated by an operations team including a centre manager, flagship officers, a community engagement officer and a communications officer (all part-time). The UoN Human Research Ethics Officer confirmed that approval from a human research ethics committee was not required for this impact analysis.

### Procedure

HCRA commenced in 2012. Regular (annual or biannual) extraction of academic outputs for HCRA members (primarily publications and research funding) from records held by UoN was completed between 2014 and 2021, within the period of the CINSW funding. Guidance on the application of FAIT was provided by author SR, a specialist in impact assessment and an independent assessor. FAIT includes three validated methodologies for assessing impact: 1) metrics based on Modified Payback [15, 19], 2) economic analysis; and 3) narrative case studies. All methods were underpinned by a Program Logic Model (PLM).

### Program logic model

**Fig. 1:**
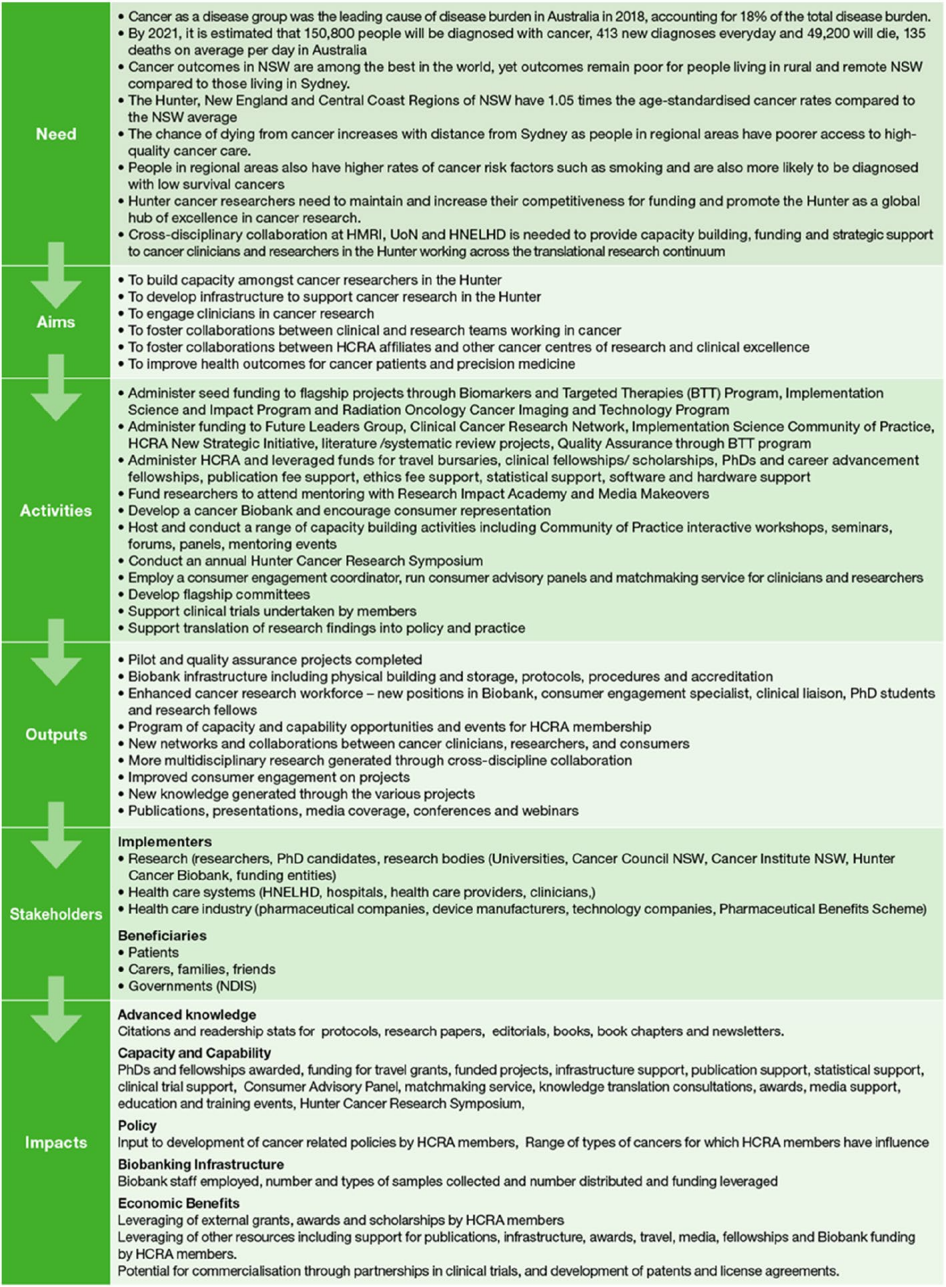
HCRA Program Logic Model

In 2021 a retrospective PLM was developed by HCRA staff facilitated by HMRI health economists. The PLM captured the ‘need’ in the community that the HCRA sought to address. It summarised the activities of the HCRA that were designed to address that need, the outputs from those activities, and how those outputs were translated to end-users. Finally, the PLM identified the impact (academic and non-academic) that was generated as a result of research translation. These impacts were grouped into domains of benefit. The design of the PLM was based on HCRA documents (e.g. CINSW funding guidelines, CINSW funding applications, HCRA strategic plans, reports to funders) and data that had either been collected by the HCRA operations team or was available from UoN administrative records. Despite some pre-2020 planning and collection of relevant impact data, the application of FAIT was considered retrospective in that the PLM was based on actual pathways that had been followed rather than a prospective PLM that would map the intended pathway at the start of the program. The PLM is presented in Fig. 1.

### Modified payback

A modified payback method, replacing qualitative statements with quantitative metrics, was used to assess impact in four domains which relate to the agreed mission and purpose of HCRA:

- Knowledge advancement (e.g. published work, newsletters and social media)
- Building capacity and capability (e.g. completed PhDs, fellowships, funded projects, software, statistical analysis, clinical trials, consumer involvement in research, education, training)
- Policy and practice change
- Infrastructure (e.g. collection and distribution of samples)

Suitable metrics were selected to reflect the types of impact that related to the mission and purpose of HCRA. Attributing the degree to which HCRA funding contributed to certain outputs and impacts (e.g. publications and leveraged grants) is inherently complex and partly subjective. Asking researchers to make this attribution retrospectively for 7 years’ worth of outputs posed an unreasonable burden. Therefore, the numbers and dollar values are presented in their entirety with a sensitivity analysis to indicate that the true attribution would likely be somewhere in the reported range.

### Economic analysis

A cost–benefit analysis to determine social return on investment is the “gold standard” for economic analysis using FAIT. This analysis relies on reporting the cost and benefits in a single unit: dollars. However, such analysis was not appropriate in this instance because many of the HCRA benefits are still being realised and those that have been realised could only be monetised with substantial assumptions. In this study a cost-consequence analysis (CCA) was used. CCA typically presents the costs of the research (in this case the cost of HCRA) and compares these against the known consequences of that funded research where the consequence is reported in either its natural unit or monetised value, if that monetary value was directly calculatable [20].

### Measuring costs

Direct research costs were captured by the HCRA operations team and verified by the centre manager. The funding for direct costs were captured via three funding sources that contributed to HCRA operations: i) CINSW under its TCRC Scheme, ii) the UoN Priority Research Centre funding scheme and iii) the NSW Health Medical Research Support Program funding administered by HMRI. The direct costs of HCRA were funds spent on the salaries of operations staff, salaries for biobank staff, other salaries (e.g. clinical fellowships, director backfill), annual cancer research symposium, event sponsorship, travel (including travel funds to present research), and purpose-specific funding granted to members (support for pilot projects, systematic reviews, research collaboration site visits, PhD student scholarship top-ups, publication fees, statistical analysis, small equipment, achievement awards). Funding granted to members primarily involved competitive processes. All salary values provided included oncosts and standard university overheads. Indirect costs such as in-kind contributions of time by HCRA members who sat on funding decision panels or flagship committees and implementation costs such as HCRA member time to participate in capacity building opportunities were not captured during the 7-year period and therefore excluded from the analysis. Additionally, involved consumers were not renumerated for their time for HCRA activities and their in-kind contributions were not captured or included in analysis.

### Valuing consequences

The consequences from HCRA comprise both non-monetary (e.g. knowledge gain, increased capacity or capability, policy change and biobanking infrastructure) and monetary benefits (e.g. funding leveraged). Non-monetary consequences, expressed in their natural units are captured via the Payback metrics and not duplicated within the economic analysis. Instead, the CCA only presents the monetised consequences of the HCRA. Some values such as leveraged biobank salaries, leveraged PhD scholarships and leveraged travel grants received 100% attribution to HCRA, i.e. they would not have occurred in the absence of HCRA. In contrast, Category 1 and 2 external grants (considered the top tiers of publicly funded competitive grants in Australia), fellowships and awards were allocated a conservative attribution of 25% with a sensitivity analysis applied to capture potential minimum and maximum values.

Economic data was collected by year so where appropriate, values were converted into 2021 dollar values based on the implicit price deflator obtained from the Australian National Accounts: Expenditure of Gross Domestic Product [21].

### Narrative

To describe the impact of HCRA, two case studies were developed that embody some aspects of the HCRA collaboration. The first describes how HCRA funding provided to an early career discovery science researcher (ECR) helped establish the ECR’s own research team and make a ground-breaking cancer discovery. The second case study showcases the HCRA Consumer Advisory Panel (CAP) and its impact on research undertaken by HCRA members. Qualitative data for both case studies were obtained by HCRA operational staff from existing documentation such as conference presentations and interviews with relevant HCRA members.

## Results

### Modified payback

Table 1 presents the results from the application of the modified Payback method of assessment, grouped within domains of benefit: ‘Advance Knowledge; ‘Capacity and Capability’; ‘Policy’, ‘Biobanking Infrastructure’ and ‘Economic Benefits’. Within knowledge advancement the HCRA produced a total of 320 weekly newsletters to keep members informed of capacity building activities, grants, and other opportunities available and created a substantial Twitter presence with between 3400 and 25,000 impressions per month.

**Table 1:** Results of Modified Payback Method of Assessment and Non-Monetisable Consequences. HCRA Impact metrics by Payback domains

Domains	Sub-categories	Metric	Result
**Advance knowledge**	**Publications, books and published presentations**	No. of cancer-related articles published by HCRA members in peer-reviewed journals including original research, protocols and editorials (total)	3822
		No. of citations (published papers)	75,736
		Average no. of citations per paper	20
		No. of book chapters authored	84
		No. of books authored	6
		No. of citations of book chapters	58
	**Newsletters/ Reports**	Weekly rapid-fire newsletters to HCRA membership	320
		No. of subscribers	567
		Quarterly Connect newsletters	32
		No. of subscribers	697
		No. of reports	2
	**Social media**	Tweets over HCRA lifetime (since June 2013)	1585
		Twitter followers	720
		Approximate Tweet impressions per month	3400–25,000
		No. of Facebook followers	405
		No. of Facebook posts	58
		No. reached per Facebook post	50–4400
**Capacity and capability**	**PhDs**	No. of PhD candidates receiving Top Up funds	23
		No. of PhD candidates supported via leveraged UoN scholarships	13
	**Fellowships**	No. of HCRA clinical fellowships offered	10
		No. of leveraged career development fellowships administered	4
		No. of leveraged career advancement fellowships administered	5
	**Travel grants**	No. of HCRA sponsored travel grants	118
		No. awarded international travel for educational exchange	4
		No. of leveraged travel grants administered by HCRA	11
	**Funded projects**	No. of HCRA funded projects and systematic reviews	85
	**Infrastructure support**	No. of HCRA projects/researchers awarded infrastructure support	35
		No. of leveraged software licenses and subscriptions funded	5
	**Publication support**	No. of publications financially supported by HCRA (e.g. systematic review salary support)	9
		No. of publications receiving leveraged financial support administered by HCRA (e.g. publication costs)	16
	**Statistical support**	No. of projects receiving HCRA funded statistical support	40
	**Consumer Advisory Panel**	No. of projects received advice from consumer panel	33
	**Matchmaking service**	No. of clinicians and researchers matched	39
	**Knowledge translation consultations**	No. of researchers who received support for a knowledge translation consultation	3
	**Future Leaders Group (FLG) Awards**	No. of recipients of leveraged FLG Awards	12
	**Media support**	No. of leveraged Media Makeover packages awarded (FLG Funds)	4
	**Education and Training Events**	No. of HCRA sponsored events	6
		Total no. of capacity building events	85
		No. of seminars	32
		No. of workshops (including STATA, Behaviours Change Wheel, Twitter, Knowledge Translation, Impact planning and metrics, new NHMRC structure etc.)	18
		No. of Conferences	16
		No. of training courses	7
		Mentor breakfasts (no. of mentors and no. of mentees)	8 & 17
		No of panels	5
		No. of ‘Shut up and Write sessions’ for FLG	6
		No. of other capacity building events	6
		No. of Consumer & Community Involvement for Researcher training courses	2
		No of individual attendance	3198
	**Hunter Cancer Research Symposium**	No. of symposiums organised	7
		No. of keynote presentations	16
		No. of invited oral presentations	76
		No. of competitively chosen rapid-fire oral presentations	70
		No. of competitively chosen poster presentations	249
	**Clinical trials**	No. of clinical trials developed by or with input from HCRA Funded CINSW-defined Category 1 or 2 members	93
		No. of clinical trials partnered with a commercial entity	11
		No. of participants recruited across 93 trials	68,784
**Policy**	**Policy change**	No. of policies and guidelines developed by or with input from HCRA Funded CINSW-defined Category 1 or 2 members	78
	**Cancer coverage**	No. of cancers covered (gastrointestinal, lung, brain, head and neck, breast, oesophageal, rectal, bladder, prostate, endometrial, thoracic, colon, lymphoma, pancreatic, Hodgkin lymphoma, skin, liver and spinal)	18
**Biobanking infrastructure**	**Samples collected**	No. of tissue samples collected	13,017
		No. of blood samples collected	38,139
		Total samples collected	51,156
	**Samples distributed**	Total samples distributed	13,691
		Tissue cases (sections or cores)	3453
		IHC slides	8456
		Bespoke tissue microarrays developed	70
		Blood aliquots	2193
		No. of projects with samples distributed	164
	**Staffing**	No. of full-time equivalent staff funded by HCRA	17
**Economic benefits**	**Grants, awards and scholarships leveraged by HCRA members**	No. of cancer-related grants, awards and scholarship top ups leveraged by HCRA members	445
		Value of leveraged funds (25% attribution)	$38,892,933
	**Other resources leveraged by HCRA**	Value of Biobank funding leveraged	$ 3,126,860
		Value of PhD funding	$ 1,227,843
		Value of fellowships	$ 514,274
		Value of travel grants	$ 13,560
		Value of publication support	$ 52,943
		Value of infrastructure support	$ 7131
		Value of FLG awards	$ 5920
		Value of media packages	$ 1998
	**Commercialisation potential**	No. of clinical trials that have partnered with a commercial entity	11
		No. of patents developed (No. 35237413)	1
		No. of license agreements	1

The largest domain of benefit from HCRA activity was in building capacity and capability, which aligns with the fact this was the largest area of investment designed to enable HCRA members to compete for research resources and opportunities that would directly benefit the local community. This capacity building can be viewed as scaffolding to enable HCRA members in their research endeavours. HCRA offered: statistical support (*n* = 40); research translation support (*n* = 8); travel grants to enable members to develop new skills (*n* = 129); clinical trials support (*n* = 12); matchmaking services to advance collaboration between researchers and clinicians (*n* = 39) and a consumer panel to ensure trained community members had input into research projects including facilitating the co-creation of research (*n* = 33 projects with consumer involvement).

HCRA members had input into 79 clinical practice guidelines covering 18 cancers. HCRA members also created an ongoing self-sustaining biobank (Hunter Cancer Biobank, now known as NSW Regional Biospecimen and Research Services) with a collection of over 51,000 samples and a throughput of 13,691 samples distributed for use in research during 2014–2021. There was also significant commercial impact including 12 clinical trials partnered with a commercial entity which generated 1 patent and 1 product license. The longer-term impact from these outputs includes potential revenue and profit streams.

### Economic analysis

The value of the investment in HCRA from 2014 till 2021 converted to 2021 values was approximately $9.7 million; with the bulk of the funds coming from CINSW ($AUD8.75 M) and the remainder coming from the HMRI Cancer Research Program and the UoN PRC CaRIT. Of this figure, 50% was allocated to operational staff salaries and a further 20% to Hunter Cancer Biobank staff salaries. Six percent was allocated to non-salary operational expenses including travel and consumables and the remaining 24% to project-based expenses and support (e.g. equipment, statistical analysis funding) (see Table 2 for expenditure).

**Table 2:** HCRA Expenditure from 2014 to 2021 adjusted to 2021 values

Cost	2021 Adjusted Value ($AUD)
Operational salaries	4,860,018
Biobank salaries	2,007,512
Project-based expenses	2,239,793
Non-salary operational expenses (including travel)	588,833
Total	9,696,155

*AUD* Australian Dollars

Monetised consequences are included in Table 3 (non-monetisable consequences are listed in Table 1). The largest item was the Category 1 and 2 grants leveraged by HCRA members over the 7-year timeframe. Although there were on average approximately 270 members at any one time, these data were collated from 100 of the more active HCRA researchers, hence Table 3 does not represent all leveraged grants. Assuming HCRA support to members only accounted for one-quarter of the leveraged grants, this would amount to $38.9 M (s.a. $15.6 - $155.6 M). HCRA was also able to leverage funds from external sources for biobank research, PhD scholarships, fellowships, travel and leadership grants, publication and infrastructure support and media packages, all of which have been assumed to not occur without the activities of HCRA (Table 3).

**Table 3.**
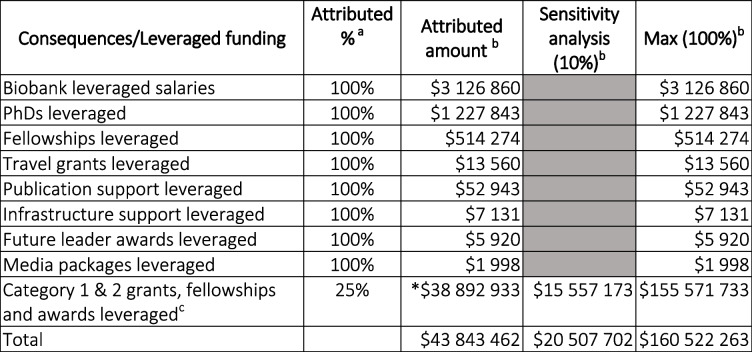
HCRA monetised consequences from 2014 to 2021 adjusted to 2021 values

^a^100% of the first eight items were attributed to the HCRA as it was determined that none of this funding would have been leveraged without the existence of HCRA

^b^All value in Australian Dollars, adjusted to 2021 value

^c^Other categories of funding were not collected for this analysis

Sensitivity analysis not required for these items

The initiation of the Hunter Cancer Biobank (now NSW Regional Biospecimen and Research Services) resulted in benefit to the research and clinical community and hence attracted an additional $3.1 million in research funding. The Biobank continues to operate independently at a similar level of throughput following completion of HCRA funding in 2021.

Acknowledging that HCRA was one of several contributors to the success of HCRA members, for the $9.7 M invested in HCRA over 7 years the conservative overall consequence that could be monetised was $43.8 M (s.a.$20.5 M - $160.5 M). Attracting this level of funding to a regional location also has the added benefit of increasing skilled jobs and economic activity.

### Narrative analysis

#### Hunter Cancer Research Alliance (HCRA) narrative

The narrative of the HCRA (Table 4) summarises the pathway from need for the program through to impact, as depicted in the PLM (Fig. 1). The narrative provides the context against which the results from the Payback metrics and economic analysis can be interpreted.

**Table 4:** Narrative of HCRA

*Background and need* People living in rural and remote NSW were identified as having higher rates of cancer and poorer cancer outcomes compared to people living in metropolitan Sydney, NSW. People in regional areas also have higher rates of cancer risk factors such as smoking and are also more likely to be diagnosed with low survival cancers. Improving both research capability and translation of research findings into practice is acknowledged as one of the pathways to improving cancer outcomes.	
*The Response* The Cancer Institute NSW initiated funding for six TCRCs in NSW which were established during 2010–2012. Funding was granted to one centre based in regional NSW, which became the Hunter Cancer Research Alliance (HCRA) in 2014, serving the HNE region. HCRA aimed to provide capacity building, funding and strategic support to cancer clinicians and researchers working across the translational research continuum in the HNE region. During 2014–2021 the CINSW provided $9.7 M in funding to support the work of HCRA.	
*Key activities* The HCRA Executive (a multi-disciplinary team of researchers, clinicians and key stakeholders) administered the CINSW funding under a single umbrella supported by three Flagship Committees, The Future Leaders Group, The Consumer Engagement Committee and Consumer Advisory Panel (CAP). The operations team provided critical support, increasing the ability of HCRA members to focus on research activities rather than administration and navigation.	
*Key outputs* HCRA funded salary and other support for 13 PhD candidates, 10 clinical fellows and 5 career advancement fellows. HCRA funding to members included funding for 85 pilot projects, 118 travel grants, 40 grants for statistical support and 35 infrastructure grants building infrastructure in regional NSW. HCRA supported the establishment of the Hunter Cancer Biobank which collected over 51,000 samples and distributed over 13,600 tissue or blood samples during that time. By 2021 the Hunter Cancer Biobank was largely self-sustaining and it continues to support many projects. See https://www.biobank.org.au/.. HCRA conducted 85 capacity-building events, seven annual Hunter Cancer Symposia. Over time the symposia were increasingly inter-disciplinary, demonstrated growing engagement from consumers and included community-facing public lectures.	
*Impacts* HCRA members leveraged 445 cancer-related grants, awards, fellowships, and scholarship top ups to a value of $38.9 M (25% attribution). Members produced new knowledge including: • The identification of stem cells necessary for endometrial cell regeneration, which has major ramifications for developing new approaches to controlling endometrial cancer incidence, with patents granted for these discoveries [22]. • Revealing the relationship between neural innervation in tumours and how this information can be used to significantly slow tumour growth [23]. This provides a new paradigm for the treatment of all tumour types that rely on nerve cell innervation and drugs based on this work are in clinical development. • Development and implementation of the world’s first real-time patient treatment verification system (Watchdog) [24–26]. The Watchdog system uses imaging devices to check in real time that the treatment is being delivered correctly, which translates to improved care for all radiation therapy patients. • Demonstrating the effectiveness of the ‘EAT’ psychological intervention [27] to improve nutrition and outcomes in people experiencing radiotherapy for head and neck cancer. Improved nutritional status, fewer treatment interruptions, lower depression scores, and higher quality of life were found. The approach has been implemented at multiple Australian sites with significant international interest. • Developing effective interventions to reduce smoking rates among priority groups such as Indigenous women, drug and alcohol users and rural people. Our researchers collaborated with the Cancer Council NSW to deliver the Tobacco Treatment Program to over 150 Social and Community Services across NSW reaching over 2000 smokers from disadvantaged groups. • The development of 79 cancer-related policies and guidelines which had input from HCRA-funded members during the period of HCRA funding. While the HCRA cannot claim full credit for these impacts, the research teams working on these different projects worked closely with HCRA and received various types of support.	

In addition, two case studies have been included to illustrate the impact of HCRA support and activities at the researcher level: Case study 1 describes how HCRA funding provided to an ECR over a 7-year period (2014–2020) helped establish an independent research team. The case study is based on a 2021 presentation by the researcher. Case study 2 illustrates some of the activities and impact of the CAP.

**Table:** 

**Case study 1** ***Background and Need***: Early career researchers face a very difficult path to establishing an independent research career, particularly if they have significant absences from research due to parental responsibilities. ***The HCRA response***: During 2012–2020 the early career researcher and their team were provided with $278,078 of support from HCRA. The support was in the form of 13 separate competitive grants. The researcher also received peer support and mentoring by participating as a committee member in the Biomarkers and Targeted Therapies and Implementation Flagship committee. ***Outcome and Impact***: The researcher reported that the salary support (a PhD scholarship for the ECR’s student and two short-term fellowships for the ECR) over an extended period of time facilitated establishment of an independent research team. The salary support also enabled the researchers to remain at a regional institution rather than them having to leave to find employment elsewhere. The travel support and funding for statistical analysis allowed the team to present new data and develop collaborations, which was critical to their progress and cohesion as a team. HCRA support was also credited with “enabling the team to thrive, keep up momentum, and establish clinical relevance” via infrastructure support, publication fees and pilot project funding. As a result the researcher’s work has revealed important information regarding how modification of a key tumour suppressor gene affects the response to DNA damaging therapy, which may result in decreased sensitivity to these therapies. The researcher was also given opportunity to build leadership capability through contribution to the wider membership of HCRA through roles such as • Membership and leadership on multiple committees, including flagship and conference organising committees. • Presenter, session chairperson and abstract marker at multiple HCRA annual symposia The ECR secured $1,987,392 in grant funding between 2014 and 2021, published 29 journal articles and supervised 6 PhD candidates and one Honours student to completion.

**Table:** 

**Case study 2** ***Background and Need***: Consumer involvement in research is increasingly valued for guiding research efforts towards outcomes which benefit the community. However, developing the skills and devoting the time required to initiate and maintain ongoing, value-adding connections with consumers poses a challenge for many researchers ***The HCRA Response:*** The Consumer Advisory Panel (CAP) was initiated in 2016. The CAP comprised members of the public who had experienced cancer either via a personal diagnosis or being a carer for a person diagnosed with cancer. At 30 June 2021, there were 32 active CAP members. Through the CAP, researchers could connect with trained and networked cancer consumers. Consumers were involved in setting the research agenda through their inclusion on HCRA grant panels, in strategic planning workshops, and as members of the Consumer Engagement Committee. Consumers joined panels for Public Lectures and Symposia organising committees, and provided direct mentoring through feedback to ECRs at mini symposia for a lay audience. Researchers could also request an introduction to a relevant CAP member to join their research team (partner) or conduct a specific activity such as testing a survey or reviewing participant information materials. CAP members were offered reimbursement for out-of-pocket expenses and were provided catering at events. ***Outcomes and Impact:*** By the end of 2018, the CAP had eight members who had completed formal consumer research training and 11 HCRA research teams had been partnered with CAP members. Consumer involvement was integrated into HCRA processes such that CAP members were invited each year to the Hunter Cancer Research Symposium. In 2019 HCRA’s annual public lecture included a presentation by consumer advocacy group Cancer Voices NSW and was attended by local State member of parliament, Sonia Hornery MP. In 2020 a CAP member (author SL) was employed as Community Engagement Officer to administer HCRA’s consumer infrastructure, mentor CAP members, and build the capacity of researchers to work with consumers. On World Cancer Day, 4 February 2020, HCRA partnered with HMRI to present an insight into current cancer research in the Hunter and the impact on patients. Over 60 community members attended the event at HMRI. In 2020, despite the COVID-19 pandemic, eight consumers were linked to six new research projects, with four as consumer partners on funding applications. From 2020, a consumer was an active member of the Symposium Scientific Committee and ‘Consumers Included’ certification was granted by Cancer Voices NSW. The 2020 Symposium included a ‘Consumers’ Choice’ award and concluded with a Consumer Involvement session: “A consumer is more than their cancer experience”. While it is not possible to quantify the number of grants that have been successful due to the involvement of consumers in applications, multiple HCRA members reported that their CAP consumer involvement was critical to their success.	

## Discussion

There is growing need to demonstrate and report the benefits from investments into health and medical research that flow back into the community. Typically, these benefits have been documented in academic terms: knowledge gained, papers published and grants leveraged. Funding bodies, the broader community, and researchers themselves have an interest in understanding how research investments generate benefits that exceed academic achievements. The FAIT impact assessment methodology was used, in this instance, to better understand the outputs from the HCRA and the many benefits and impacts- particularly to cancer research and cancer researchers in a rural and regional location of Australia. Benefits were found in all five of the domains of benefit identified as being applicable to the aims and purpose of the HCRA (internal report), particularly in capacity building of next generation researchers through a series of capacity building activities, and by leveraging an estimated $AUD43.8 million funding available to support the ongoing career development of regionally based early and mid-career cancer researchers. This leveraging of significant additional research funding in such a short timeframe also indicates the efficacy of HCRA in supporting members to attract substantial research funding to a regional area of Australia. HCRA funded 85 pilot projects, disbursed 118 travel grants, provided 40 grants for statistical support, and supported 35 infrastructure grants to build research infrastructure in regional NSW.

Perhaps the best example of valuable capacity-building to arise from the HCRA is creation of the NSW Regional Biospecimen and Research Services, or ‘Biobank’. With foundational funding and infrastructure support from the HCRA, during this time the biobank collected over 51,000 samples and distributed over 13,600 tissue or blood samples. The biobank is now a standalone, self-supported entity that continues to deliver translational biomedical research infrastructure and services to the Hunter cancer research community and beyond.

Given the lengthy 17–20 years [28, 29] it is estimated to take for research evidence to reach clinical practice, it was also encouraging to be able to show, after a significantly shorter timeframe, some changes in cancer practice as a result of research conducted under the auspice of the HCRA. This includes the implementation of ‘Watchdog’ and ‘EAT’ as described in Table 4. This evidence of HCRA supported research translation demonstrates that the HCRA was able to fulfil its aims of engaging and collaborating with clinicians in research to improve outcomes for cancer patients and precision medicine. Further, the HCRA proved commercialisation potential with members producing 11 commercially partnered clinical trials, 1 registered patent, and 1 licensing agreement. To have achieved these outcomes in a relatively short timeframe supports the potential for future schemes to focus on the commercialisation of research findings. HCRA funding or HCRA-leveraged funding also provided salary support for 13 PhD candidates, 10 clinical fellows and 5 career advancement fellows, all in regional NSW, and all potential future cancer research leaders. Additionally, in this narrow timeframe HCRA members collectively collaborated on and produced 3822 publications.

To the authors’ knowledge, no impact analyses of this type and scale have been undertaken by any of the other CINSW-funded TCRCs. Studies of the impact of large research centres in general is lacking. Upon funding the TCRCs, the CINSW identified that “the key objective (was) to facilitate closer collaboration between researcher and clinician to drive the generation of practice-improving research and its more rapid adoption for improved patient outcomes” [30]. In addition to a high level of involvement in governance by HNELHD clinicians, the HCRA was able to foster ongoing relationships between researchers and clinicians especially through the hosting of the annual HCRA Symposium. The symposium gave early-career researchers and early-career clinician-researchers a platform to showcase their ideas and open the dialogue for collaboration, leading to several successful clinical trials as reported in the results. Over time, the symposia were increasingly inter-disciplinary, demonstrating a growing engagement from consumers, in addition to the engagement enabled by a dedicated consumer program within HCRA.

### Limitations

The counterfactual to the existence of HCRA – what would have happened if HCRA did not exist – is an important question that cannot be answered by this impact analysis. However, as a potential indicator of the effect of HCRA, we examined the productivity of the primary researchers listed in the 2011 application to form the Priority Research Centre for Cancer who also remained part of HCRA up to 2021 (*n* = 9). Five years pre-HCRA (2009–2013) was compared to 5 years during-HCRA (2017–2021) for research income, peer-reviewed journal publications and PhD completions for the nine researchers. Of these 27 indicators of research productivity, 17 indicators increased from pre-HCRA to post-HCRA and 10 either did not change or decreased, suggesting that HCRA may potentially be associated with increased research productivity. In addition, data collection for compiling research outcomes for the FAIT analysis was burdensome to members and may not present the full picture of impact, particularly regarding leverage estimates, proportional attribution of outputs and presentations.

The direct monetised consequences from HCRA were limited because HCRA was mainly a research collaborative and translational research centre focused on capacity building and research translation, with few research projects being funded directly from its resources. Impact from access to a cancer biobank is still being realised and difficult to monetise. Hence, this impact assessment of HCRA captures a snapshot of research gains at a point-in-time while the downstream, longer-term impacts such as commercialisation potential are still unfolding. While many HCRA-related projects did focus on factors relevant to poor health outcomes in the Hunter region (e.g. preventive behaviours), it is unlikely that direct attribution of the health impact of HCRA research on the local population could be reliably estimated at this time. The economic analysis that included attribution of HCRA contribution was necessarily conservative, which may have downplayed the success of HCRA funded activities. Nonetheless, the implementation cost of delivering HCRA activity was not able to be fully captured and is likely to be greater than the $9.7 M estimated.

## Conclusions

The use of FAIT facilitated an understanding of the significant impact and downstream benefits that can be achieved by the provision of infrastructure-type funding to regional and rural research collaborations to help address inequities in research activity and health outcomes.

### Supplementary Information

**Additional file 1: Supplementary Figure.** Region covered by Hunter New England and Central Coast Local Health District.

## Data Availability

The datasets used and/or analysed during the current study are available from the corresponding author on reasonable request.

## Abbreviations

AUD: Australian Dollars
CAP: Consumer Advisory Panel
CINSW: Cancer Institute of New South Wales
CCA: Cost-consequence analysis
ECR: Early career researcher
FAIT: Framework to Assess the Impact from Translational health research
FLG: Future Leaders Group
HCRA: Hunter Cancer Research Alliance
HMRI: Hunter Medical Research Institute
HNE: Hunter New England
HNELHD: Hunter New England Local Health District
NSW: New South Wales
PLM: Program Logic Model
PRC CaRIT: Priority Research Centre for Cancer Research, Innovation and Translation
TCRC: Translational Cancer Research Centre
UoN: University of Newcastle
