# Supplementary material for: The impact of a regionally based translational cancer research collaborative in Australia using the FAIT methodology
Source: BMC Health Serv Res. 2024 Mar 11;24:320. doi: 10.1186/s12913-024-10680-2 (PMC10926601; doi:10.1186/s12913-024-10680-2)

**Supplementary Figure**: **Region covered by Hunter New England and Central Coast Local Health District**


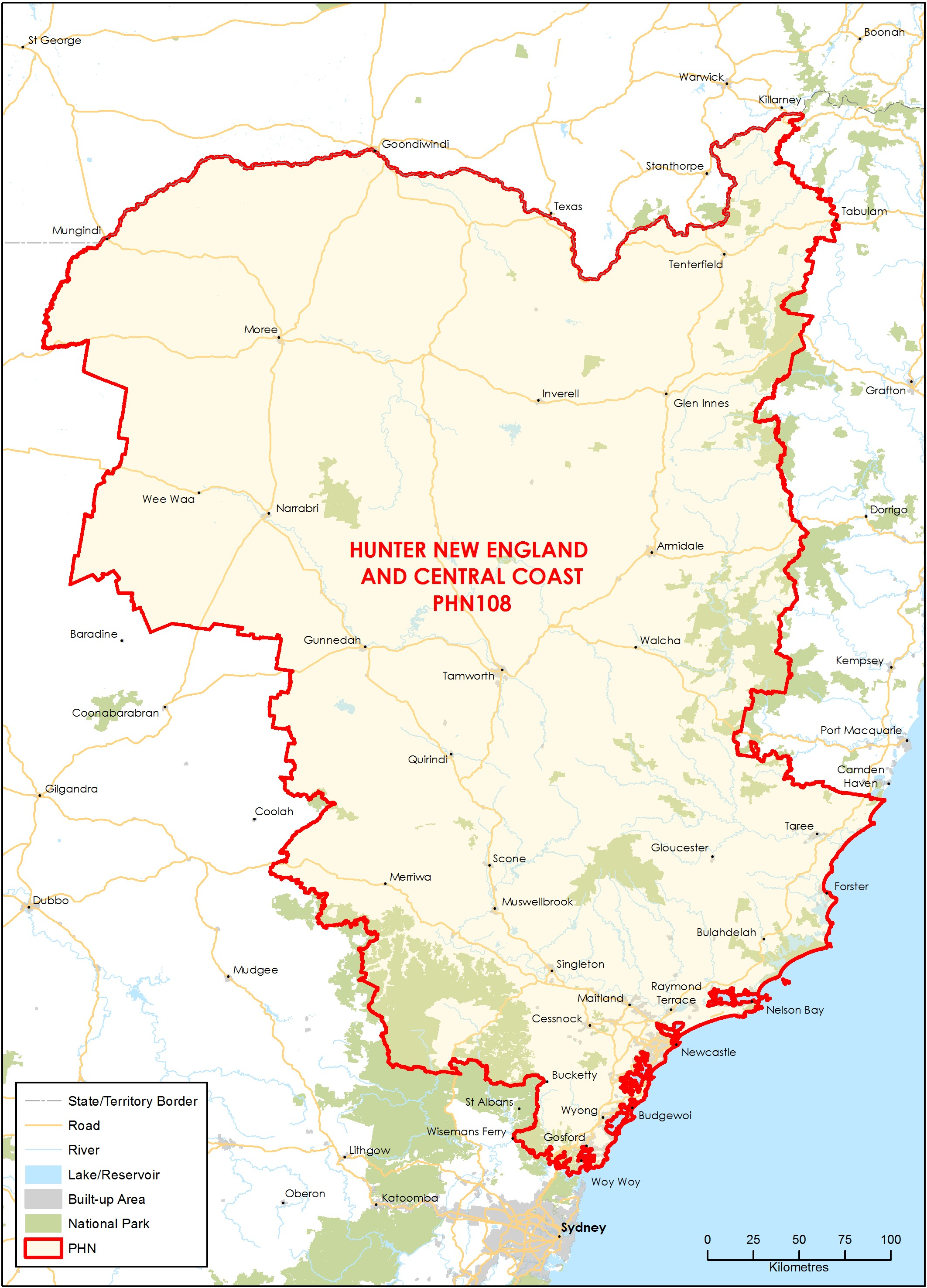

Supplement: Supplementary file 1 — Additional file 1: Supplementary Figure. Region covered by Hunter New England and Central Coast Local Health District. [file 12913_2024_10680_MOESM1_ESM.docx]
